# Malaria control in Malawi: are the poor being served?

**DOI:** 10.1186/1475-9276-6-22

**Published:** 2007-12-02

**Authors:** Don P Mathanga, Cameron Bowie

**Affiliations:** 1 Department of Community Health, College of Medicine, University of Malawi, Blantyre, Malawi; 2 Malaria Alert Centre, College of Medicine, Blantyre, Malawi

## Abstract

**Background:**

In Africa, national governments and international organizations are focusing on rapidly "scaling up" malaria control interventions to at least 60 percent of vulnerable populations. The potential health and economic benefits of "scaling up" will depend on the equitable access to malaria control measures by the poor. This paper analyses the present inequalities in access to malaria interventions in Malawi.

**Methods:**

Equity in access to malaria control measures was assessed using the Malawi Demographic Health Survey (DHS) 2000 and the 2004 national survey on malaria control. Utilisation of malaria control methods was compared across the wealth quintiles, to determine whether the poor were being reached with malaria control measures.

**Results:**

Overall ITN coverage increased from 5% in 2000 to 35% in 2004. However, there was a disproportionate concentration of ITNs amongst the least poor compared to the poorest group. Effective treatment of fever remains unacceptably low with only 17% of the under-five children being promptly treated with an effective antimalarial drug. And only 29 percent of pregnant women received the recommended dose of at least two doses during the pregnancy. No income related inequalities were associated with prompt treatment and IPT use.

**Conclusion:**

The present distribution strategies for ITNs are not addressing the needs of the vulnerable groups, especially the poor. Increasing access to ITNs by the poor will require innovative distribution models which deliberately target the poorest of the poor.

## Background

The global community already has highly effective tools to control malaria, namely: insecticide-treated nets (ITNs), intermittent preventive treatment (IPT) in pregnant women, and prompt and effective case management. Under trial conditions, ITNs have consistently led to a 15 to 20 percent reduction in child deaths [1]. Evaluations of programmes delivering ITNs have also shown substantial benefits from their use [2]. IPT has also been shown to reduce malaria and its consequences – placental parasitemia and anemia – in the pregnant woman and to reduce the risk of low birth weight in newborns [3]. And finally, prompt and effective case management is associated with reduced anemia in young children [4] and can be lifesaving for persons with acute, severe, or complicated malaria. However, maximum benefit from these interventions can only be realized if national malaria control programs can achieve high coverage of the interventions, especially amongst the poor.

Because malaria causes poverty and prevents or reduces people's ability to escape poverty, and because the consequences of malaria fall heavily on the poor [5], in Malawi, these three interventions have been included in the Essential Health Package (EHP), the health sector's contribution to poverty reduction. As a result, the national malaria control program is focusing on the scale up of the three interventions so as to significantly reduce malaria morbidity and mortality. However, the success of these efforts will be limited if the poor fail to access malaria control measures.

This study analyses the inequalities in access to malaria interventions in Malawi and will provide the baseline for the monitoring and evaluation of equitable access and use of malaria interventions. It is one of a series of studies commissioned by the Malawi Ministry of Health (MOH) through its Sector Wide Approach (SWAp) Technical Working Group on Monitoring and Evaluation. Findings from this study will also provide practical recommendations for the implementation of the essential health package.

## Methods

The data came from two national data sets, including the Malawi Demographic Health Survey (DHS) of 2000 and the 2004 national survey on malaria control. We reanalyzed the data sets with emphasis on evaluating the equitable coverage of malaria interventions by socioeconomic status (SES). SES was defined based on household assets (such as radio, bicycle, car, television, type of roofing, floor etc) reported by the survey respondent. Each asset was assigned a weighting value, using principal component analysis as described by the World Bank and Measure published methods [6]. A household was assigned a standardized score for each owned asset, where the score differed depending on whether or not the household owned that asset. For each household, these scores were summed and households ranked into five wealth quintiles. Utilization of malaria control methods was then compared across the wealth quintiles to determine whether the poor were being reached with malaria control measures.

Concentration Index (CI) was used to measure income related inequality in accessing malaria intervention across all the wealth quintiles. The concentration index, as a measure of equality, ranges from -1 to +1 [7]. For this study, an index of zero indicates perfect equality and a positive or negative concentration index signifies inequality.

## Results

### Insecticide Treated Nets use

**Figure 1:**
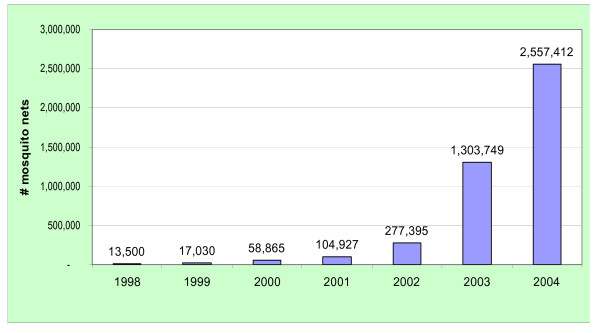
Number of ITNs distributed through the social marketing program: 1998 – 2004, Malawi.

Figure 1 shows the number of ITNs distributed every year since 1998, when the ITN social marketing program was first introduced in Malawi. By March 2004, 35% of the households in Malawi owned at least one ITN. For both the 2000 and 2004 data sets, ITN ownership was associated with living in urban areas and higher educational levels.

**Figure 2:**
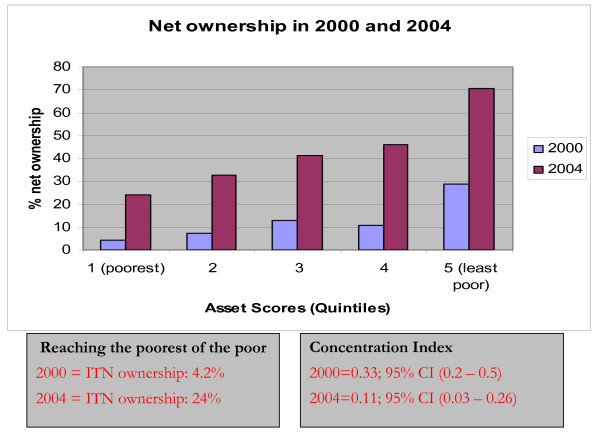
ITN ownership by wealth quintile: 2000 and 2004, Malawi.

Figure 2 shows ITN ownership across the wealth quintiles in 2000 and 2004. A positive concentration index both in 2000 [CI = 0.33 (0.16–0.50)] and 2004 [CI = 0.11 (0.03 – 0.26)] indicates the disproportionate concentration of ITN ownership amongst the least poor compared to the poorest group. Although the overall ITN coverage had increased significantly from 5% in 2000 to 35% in 2004, the corresponding decrease in concentration index from 0.33 to 0.11 was not significant as can be noted from the overlapping confidence intervals.

### Treatment of fever

**Figure 3:**
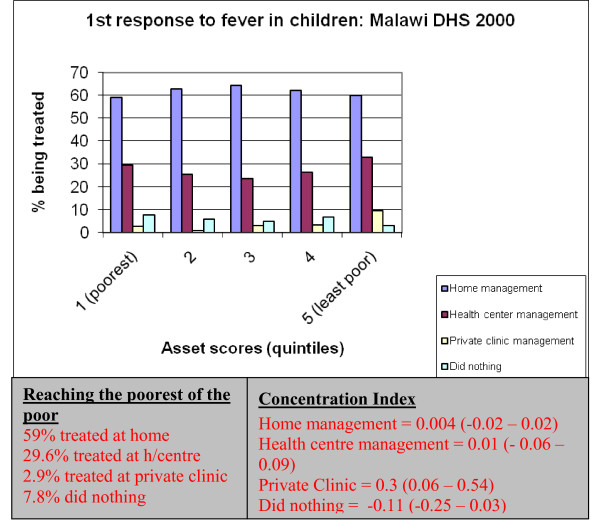
First response to fever in Malawi: Malawi DHS 2000.

Using data from the Malawi DHS 2000, home management of fever was very common, with about 60% of the cases being managed at home first, across all the socio-economic groups. The use of home management [CI = 0.004 (-0.02–0.02)] and health facilities [CI = 0.01 (-0.06–0.09)] for treatment of fever was not statistically different across the socio-economic groups (Figure 3). Not surprisingly, there was income related inequalities in the treatment of fever at private clinics [CI = 0.3 (0.06–0.54)], where the least poor group was more likely to use private clinics than the poorest group.

Only 23% of children with fever, regardless of where they received treatment, had access to an effective antimalarial drug in 2000. Income related inequalities in accessing effective drug were present with only 21.5% of children in the lowest quintile getting treatment compared to 32% in the highest quintile [CI = 0.03 (0.01–0.06)]. Only 17.6% of the children were treated promptly (use of effective antimalaria within the first 24 hours of fever), although prompt treatment was not statistically different across the wealth quintiles [CI = -0.16 (-0.59–0.26)].

### Intermittent Preventive Treatment

The 2000 Malawi DHS indicate that 68 percent of all mothers received at least one dose of Sulphadoxine-Pyrimethamine (SP)/Fansidar as a prophylaxis and that 29 percent received the recommended dose of at least two doses during the pregnancy. For those that received the recommended two doses or more the differences across the socioeconomic groups was not statistically significant.

## Discussion

Rapidly increasing national coverage of ITNs is central to Malawi's strategy of malaria control [8]. The challenge is in finding a distribution model that would ensure high and equitable ownership of ITNs. In Malawi, ITNs are distributed through the social marketing program. By definition, social marketing discriminates against the poor who may not have disposable income needed to afford health products [9]. Not surprisingly, by 2004, only 24% of households in the lowest socio-economic quintile (poorest) had ITNs compared to 71% in the least poor quintile. Alternative distribution methods are therefore urgently needed if Malawi is to scale up ITNs for impact, especially amongst the very poor. Because surveys have shown that poverty is the main reason for not owning an ITN [10], future distribution methods should include free distribution of ITNs at least to the vulnerable groups, including the very poor. There is evidence to show that targeted free distribution of ITNs is equitable. In Ghana, overall household ITN ownership increased from 4.4 percent to 94.4 percent when free distribution of ITNs was linked to a measles campaign with households in the poorest quintile achieving a post-campaign coverage ten times higher than the pre-campaign coverage of households in the wealthiest quintile [11]. Distribution of 100% subsidized ITNs to pregnant women and under-five children could also be linked antenatal and Expanded Program on Immunization (EPI) services. In fact, free distribution through antenatal clinics has been shown to be a simple, cheap and equitable approach to delivering ITNs to pregnant women [12]. However, more operational research is required to assess the feasibility of linking ITN distribution to routine EPI services.

Other observers have called for the free distribution of ITNs amongst the poorest of the poor. Unfortunately, proxies for the identification of the poorest of the poor are difficult to define under operational settings and have not been defined in Malawi. In our view, free distribution of ITNs should focus on the already identifiable vulnerable populations: pregnant women and children under five years of age. Only when very high coverage and equity is achieved in these groups, will the burden of malaria morbidity and mortality be significantly reduced.

In 2000, only 17% of children with fever were promptly treated (within 24 hours) with an effective antimalarial drug. About 60% of all the fevers were managed at home with drugs bought at pharmacies or local shops. Although there was no income related inequality across the wealth quintiles both in prompt treatment and home management of fever, the least poor group were more likely to have treated fever in children with an effective antimalaria drug than the poorest group. These statistics are more worrying because untreated or delayed treatment of falciparum malaria contributes both directly and indirectly to the death of non-immune individuals, sometimes within hours of the onset of symptoms [13]. The lack of inequality could be attributed to the fact that in 2000, the effective antimalarial drug (SP) in Malawi was locally manufactured and widely accessible at a minimal fee. However, the Government of Malawi is now changing its treatment policy to an artemisin-based combination therapy which will require visits to a health center and if available in local shops or pharmacies will be prohibitively expensive. With this new policy, it's more likely that income related inequalities will hinder access to effective treatment by the poor. It is important therefore that new distribution methods, which deliberately target the poor, be put in place to counter these potential inequalities. One such method could involve the empowerment community health workers to distribute effective treatment at community level, a method that has been proven to significantly reduce malaria morbidity and mortality in children, and to increase equity in access [14,15]. However, the establishment of such a distribution method would depend on the cost implication of the program to the country. It is our view though that a drug policy which fails to address equity issues will lead to increased malaria morbidity and mortality.

In 2000, only 29% of pregnant women had received the recommended two doses or more of SP/Fansidar during the last pregnancy. Although there was no income related inequalities across the wealth quintiles, this figure is well below the Abuja recommendation of 60% of pregnant women using IPT. In Malawi, barriers to increasing IPT include inadequate knowledge or confusion among health staff regarding the proper timing of the second dose of SP, stock shortages of SP at health facilities and apprehension among pregnant women to take SP [16]. To avoid the confusion as to when to administer the second dose, IPT could be given at every antenatal contact with the pregnant woman. To date there is no evidence that shows that women who receive more than two doses of SP have an increased adverse drug reactions or increased number of adverse pregnancy outcomes than those on the recommended two doses [17]. In the event that a combination therapy is recommended to replace SP for IPT, then further research on the effective distribution channels to ensure equitable access to IPT will be needed. The lack of inequalities in IPT could be explained by the fact that in Malawi, antenatal attendance is very high across all socioeconomic quintiles, providing a great opportunity to reach all pregnant women with IPT.

## Conclusion

Progress on implementing the effective malaria interventions described above has not been satisfactory since the Abuja Summit. Rapid scale-up of these interventions could lead to substantial improvements in child survival and in health and economic benefits. It is obvious that increasing access to ITNs by the poor will require innovative distribution models which deliberately target the poorest of the poor. Although income related inequalities were not prominent in prompt treatment of fever and IPT use, there is potential for inequalities if new expensive drugs replace Sulfadoxine Pyrimethamine.

## Competing interests

The author(s) declare that they have no competing interests.

## Authors' contributions

DPM devised and designed the study, undertook data analysis and wrote the first draft of the manuscript. CB contributed to the study design, the analysis and revising the manuscript.
